# Multiscale Entropy Analysis of Heart Rate Variability in Neonatal Patients with and without Seizures

**DOI:** 10.3390/bioengineering8090122

**Published:** 2021-09-09

**Authors:** Lorenzo Frassineti, Antonio Lanatà, Benedetta Olmi, Claudia Manfredi

**Affiliations:** 1 Department of Information Engineering, Università degli Studi di Firenze, Via Santa Marta 3, 50139 Firenze, Italy; antonio.lanata@unifi.it (A.L.); benedetta.olmi@stud.unifi.it (B.O.); claudia.manfredi@unifi.it (C.M.); 2 Department of Medical Biotechnologies, Università di Siena, 53100 Siena, Italy

**Keywords:** neonatal seizures, HRV, multiscale entropy, HIE, ECG

## Abstract

The complex physiological dynamics of neonatal seizures make their detection challenging. A timely diagnosis and treatment, especially in intensive care units, are essential for a better prognosis and the mitigation of possible adverse effects on the newborn’s neurodevelopment. In the literature, several electroencephalographic (EEG) studies have been proposed for a parametric characterization of seizures or their detection by artificial intelligence techniques. At the same time, other sources than EEG, such as electrocardiography, have been investigated to evaluate the possible impact of neonatal seizures on the cardio-regulatory system. Heart rate variability (HRV) analysis is attracting great interest as a valuable tool in newborns applications, especially where EEG technologies are not easily available. This study investigated whether multiscale HRV entropy indexes could detect abnormal heart rate dynamics in newborns with seizures, especially during ictal events. Furthermore, entropy measures were analyzed to discriminate between newborns with seizures and seizure-free ones. A cohort of 52 patients (33 with seizures) from the Helsinki University Hospital public dataset has been evaluated. Multiscale sample and fuzzy entropy showed significant differences between the two groups (*p*-value < 0.05, Bonferroni multiple-comparison post hoc correction). Moreover, interictal activity showed significant differences between seizure and seizure-free patients (Mann-Whitney Test: *p*-value < 0.05). Therefore, our findings suggest that HRV multiscale entropy analysis could be a valuable pre-screening tool for the timely detection of seizure events in newborns.

## 1. Introduction

As stated by the International League Against Epilepsy (ILAE) position paper [1], neonatal seizures represent one of the most relevant signs of a neurological insult or acute illness, with an incidence of about 1–5 per 1000 live births, and with a significantly high percentage in preterm newborns [1,2]. Although the discussion about etiology and possible causes remains open, it is well known that the majority of neonatal seizures (about 85% [2,3]) are related to hypoxic-ischemic encephalopathies (HIEs), stroke, infections, metabolic, and genetics. If not promptly detected and treated, they can strongly affect the neurodevelopment of the infant [2,4]. However, newborn seizure detection is still tricky and time-consuming, even in highly specialized settings such as neonatal intensive care units (NICUs). Due to different manifestations, ictal patterns and etiologies, their identification based only on the observation of the clinical signs is challenging [1]. Currently, electroencephalography (EEG) is the accepted gold standard often combined with synchronized video recordings (video-EEG) [5].

Research findings have shown that EEG is the basic technique for detecting neonatal seizures with no evident clinical signs for addressing the so-called electrographic-only seizures or sub-clinical seizures [1]. Nevertheless, the use of EEG or video-EEG requires expert staff available 24/24 h for proper detection and interpretation of ictal events. 

Moreover, amplitude-EEG (aEEG) or color spectral density array (CSDA) have been proposed as valid alternatives to EEG for speeding up and facilitating neonatal seizure detection. However, to date, their performance is still lower than in EEG [6,7]. For these reasons, several EEG-based neonatal seizure detectors (NSDs), including artificial intelligence techniques, have been introduced to support the physician’s decision [8,9,10].

At the same time, there has been increasing interest in investigating other signals besides EEG for neonatal seizure detection and characterization [8,11,12]. In particular, electrocardiography (ECG) might be a valid support to this task thanks to its ease of use, less invasiveness and lower costs than EEG. Therefore, ECG-based methods could be useful alternatives where EEG-related techniques are not readily achievable. 

From ECG, the heart rate variability (HRV) analysis could provide helpful information about neonatal seizures. In [13,14,15,16], it was shown that some neonatal seizures could directly or indirectly impact the newborn’s cardiac function and, in general, the cardio-regulatory system. In particular, using HRV frequency measures, in [16], it was shown that newborns with seizures might impact autonomic cardiovascular regulation. These results suggest that nonlinear HRV measures could help to untangle the complex mechanisms between neonatal seizures and the autonomic system. Information theory (IT) and nonlinear analysis methods might provide reliable information about neonatal seizure dynamics and characteristics. In particular, entropy features used for HRV analysis in the adult were promising for detecting autonomic systems variations [17,18,19]. Multiscale entropy analysis could increase the information obtained with single scale analysis about the underlying physiological process [20]. Thus, applying these findings to newborns could be usefully exploited, considering the differences between ictal events in adults and infants, both for etiology and the electro-clinical characteristics [1,21,22]. 

This study aims at evaluating whether HRV multiscale entropy features might provide augmented information for characterizing autonomic system dysregulation during neonatal seizures. Moreover, we investigated if this information could be helpful in neonatal seizure detection. In particular, the effectiveness of entropy features in the discrimination between newborns with seizure events and seizure-free ones have been explored, determining at which scale these differences become evident.

The paper is organized as follows: Section 2 describes the neonatal seizure dataset on which our methods have been tested, the computed entropy indexes, and the performed multiscale analysis. Moreover, the statistical tests performed to evaluate the differences between newborns with and without seizures are described. Section 3 shows the statistical results of HRV entropy measures at different scales. Section 4 resumes all the achieved results and comments about the usefulness of multiscale entropy to explain mechanisms behind neonatal seizures.

## 2. Materials and Methods

The proposed methods have been evaluated on a public dataset of neonatal seizures collected at Helsinki University Hospital [23]. The dataset consists of 79 newborns independently annotated by three experts. In particular, 39 newborns had a unanimous consensus between the experts about the presence of seizures events inside the recordings. Instead, for 22 newborns, the experts did not unanimously find seizure events. Thus, they were considered seizure-free patients [23]. Only the newborns with unanimous consensus across experts and for which the ECG signal was present in the dataset have been retained for analysis. For this reason, we excluded the remaining 18 newborns without unanimous consensus. Moreover, we discarded further 9 patients (6 with consensus seizures and 3 seizure-free) because the ECG signal was not present in their recordings or was highly corrupted by noise. Thus, the analysis has been performed on 33 patients with seizure events and 19 seizure-free patients. The ECG signals were recorded as the EEG channels by NicoletOne vEEG System, Natus Medical [23]. Two leads were placed on the newborn’s chest, and the ECG was acquired with a sampling rate of 256 Hz. For each ECG recordings, the inter-beat-interval (IBI) time series has been achieved by the Kubios software (Version 3, Kubios Oy, Kuopio, Finland) [24], a widely used tool for the HRV analysis, and the Pan-Tompkins’ method for IBI time series extraction [24]. Data cleaning was performed applying a first-order detrending and a medium artefact correction.

Kubios software includes a limited number of nonlinear HRV measures; therefore, we exported the IBI time series and computed all the multiscale entropy indexes and relative statistical analysis under MATLAB 2020b environment [25]. 

**Figure 1:**
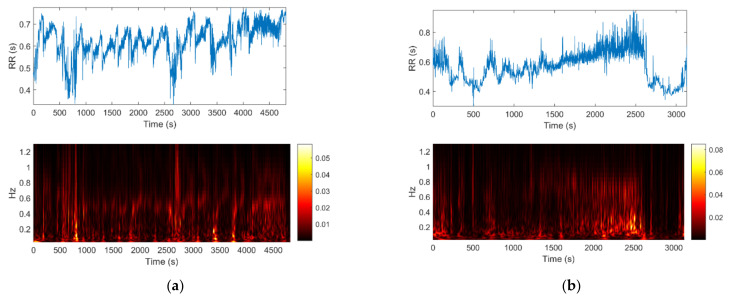
(**a**) Newborn with seizure events. (**b**) Seizure-free subject. Above: inter-beat (RR) time series extracted by Kubios. Below: the corresponding time-frequency representation using Continuous Wavelet Transform.

Two examples of RR trends are shown at the top of Figure 1: on the left, a patient with seizure events and on the right, a seizure-free subject. For each signal the time-frequency plot is shown below. The frequency range is 0.04–1.3 Hz [16].

To the untutored eye, differences between the two cases are difficult to detect by visual inspection, therefore other approaches have to be investigated, such as HRV multiscale entropy. 

The HRV entropy features were calculated on the segmented IBI time series applying a sub-windowing procedure, with non-overlapping windows lasting 4 min [15]. These procedures were repeated for each patient for the entire recording and discarding the windows that included recording pauses [23]. Furthermore, each window was labelled according to the information provided by the experts. Three classes have been selected: class “1” identifies a window with seizure events lasting at least 1s; class “int” (i.e., interictal) identifies a window without seizure events, but belonging to a patient with seizures; class “0” identifies a window without any seizure event and belonging to a seizure-free patient. Specifically, we defined “seizure windows” only for which the three experts simultaneously labelled that window as a seizure. A total of 441 interictal windows, 284 windows with seizure events, and 342 seizure-free windows have been achieved. Thus, a total of 1067 windows from the 52 patients were obtained.

For each window, the following HRV entropy measures were computed: Approximate Entropy (AE) [26]; Sample Entropy (SE) [27]; Generalized Sample Entropy (GSE) [28] and Fuzzy Entropy (FE) [29]; Permutation Entropy (PE) [30] and Distribution Entropy (DE) [31]. All the indexes were evaluated at different scales, defining a coarse-grained time series for each of them. More details are reported in Section 2.1. From Section 2.2, Section 2.3, Section 2.4, Section 2.5 and Section 2.6 are devoted to a short discussion about the basic concepts of each index.

### 2.1. Multiscale Analysis

Research findings showed that multiscale scale entropy indexes in HRV analysis are helpful both for the characterization or detection of several pathologies and the description of different dynamics of the autonomic system [17]. Recently, the multiscale entropy analysis was successfully applied to ECG signals to detect and characterize seizures in adults [17]. These results open the possibility of evaluating these techniques also in newborns. This paper implements the multiscale analysis for all the entropy indexes (except for GSE) using the mean operator’s coarse-grained procedure to generate the time series at each scale. The coarse-grained procedure is reported in Equation (1).

(1) $$y^{s}\left(j\right)=\frac{1}{s}\overset{js}{\underset{i=\left(j-1\right)s}{\sum }}x\left(i\right),\;1\;\leq \;j\;\leq \;\lfloor N/s\rfloor$$

where *y* is the coarse-grained time series, *s* is the scale factor, and *x*(*i*) is the *i*-th sample of the original time series of length *N*. The symbol ⌊.⌋ indicates the integer part of its argument.

**Figure 2:**
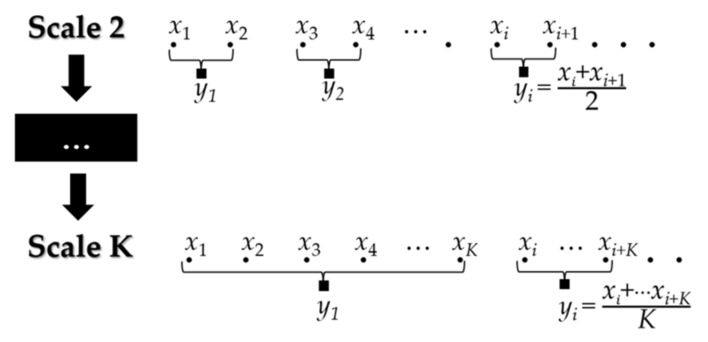
Example of the coarse-grained procedure from scale 2 to scale *K*.

Figure 2 shows the coarse-grained procedure from scale 2 to scale *K*. Notice that, at scale 1 (*s* = 1), the coarse-grained series is equivalent to the original time series. The second-moment operator (i.e., the variance) has been used to generate the coarse-grained time series for GSE computation [28]. Thus, at scale 1, the only difference between GSE and SE is the threshold parameter (for more details, see Section 2.2). Following [20] and considering the length of the used windows (4 min), we computed the coarse-grained series up to scale 6.

For HRV analysis, a 5-min window should be preferred [32]. However, as stated in [15,16,33], a standardized norm is missing for newborns. Due to the higher newborn heart rate with respect to that of adults, shorter windows are suitable. In this paper, a 4-min window is used as a compromise between a consistent estimation of multiscale HRV entropy indexes [20] and the capability to detect seizure events [33].

Thus, considering the average newborn heart rate at rest (100–200 bpm [33]) and the defined window length, we computed up to scale 6 to have at least 10^*m* points at each scale and for all the entropy indexes considered [27,34,35]. Where *m* is the embedding dimension. In this work, we use *m* = 2 for all the entropy indexes. This choice could avoid an inaccurate estimation of entropy values due to a coarse-grained scale at higher scales where the number of points becomes too low [36].

### 2.2. Approximate Entropy, Sample Entropy, and Generalized Sample Entropy

Approximate Entropy (AE) [26] represents the conditional probability that time series that are close to each other for *m* consecutive samples (i.e., the embedding dimension) will be close to each other when one or more samples are known. To introduce the definition of AE, let {*X_i_*} = {*x*_1_*…x_N_*} be a time series where *N* is the number of samples. Moreover, *u_m_*(*i*) and *u_m_*(*j*) are vectors of length *m* (e.g., *u_m_*(*i*) = {*x_i_*,…,*x_i_*_+*m*−1_} for any *i* and *j*). Let $n_{i}^{m}\left(r\right)$ be the number of vectors *u_m_*(*i*) and *u_m_*(*j*) that satisfy ${d[u}_{m}{(i),u}_{m}(j)]\;\leq \;r$, where *d* is the *∞*-norm and *r* a threshold parameter. Therefore $C_{i}^{m}\left(r\right)=\frac{n_{i}^{m}\left(r\right)}{N-m+1}$ represents the probability that a vector *u_m_*(*i*) is close to vector *u_m_*(*j*). The average of the $C_{i}^{m}$ is the probability that any two vectors are within the threshold parameter *r*. Thus, AE can be defined as in Equation (2):

(2) $$AE(m,r,N)=\frac{1}{N-m+1}\overset{N-m+1}{\underset{i=1}{\sum }}\ln C_{i}^{m}\left(r\right)-\frac{1}{N-m}\;\overset{N-m}{\underset{i=1}{\sum }}\ln C_{i}^{m+1}\left(r\right)$$

In general, low AE values are related to more predictable and regular time series, thus providing a degree of complexity and irregularity [37]. 

As a modification of AE, Richman et al. [27] proposed the Sample Entropy (SE). The main difference is that SE excludes the self-matches (i.e., *i* *≠ j*), thus reducing the bias of AE. Moreover, SE was proved to be less dependent on time series length, with a higher consistency in different contexts. To date, it is one of the entropy measures most applied to physiological analysis [17]. 

We remark that also SE may present some issues when applied to short times series or non-stationary signals, and it may give misleading results when data are oversampled and temporally correlated [38]. To overcome its limits and enhance its advantages for specific applications, several modifications of SE and multiscale SE (MSE) were proposed [17,35]. This is the case of refined MSE [39], composite MSE [40], refine composite MSE [41], and modified MSE [36] introduced to overcome possible issues related to short time series. Other variants of MSE, for example, Intrinsic Mode Entropy [42] or Hierarchical Entropy [43], were proven to detect high frequencies in signals at different scales. Finally, we point out that other indexes such as Adaptive Multiscale Entropy [44] as well as Intrinsic Mode Entropy were proved useful for non-stationary signals.

Recently, Costa et al. [28] defined the Generalized Sample Entropy (GSE) for multiscale experiments. The main difference between GSE and MSE concerns the definition of the coarse-grained time series: instead of using the mean operator, in [28], they used the variance (VAR). GSE can quantify the dynamical time series volatility properties at different scales. Concerning HRV analysis, intermittency in energy and information flows caused by abnormal GSE trends may be related to some pathophysiological processes during both cardiac cycles of activation and recovery (e.g., during depolarization and repolarization) [28]. 

For all these entropy measures, and for all the scales, we used as embedding dimension *m* = 2 as in [17,27], and *r* = 0.2 as threshold parameter of the time series standard deviation for multiscale AE (MAE) and MSE, while for GSE we used *r* = 0.05. This choice allows taking into account the different values in the amplitude of the VAR [28]. The results are reported in Section 3.

### 2.3. Fuzzy Entropy

Fuzzy Entropy (FE) can be considered as an extension of SE [29]: for a time series {*X_i_*} let *u_m_*(*i*) and *u_m_*(*j*) be vectors of length *m*, where *m* is the embedding dimension. Let ${\bar{u}}_{m}\left(i\right)$ and ${\bar{u}}_{m}\left(j\right)$ be the mean values of vectors *u_m_*(*i*) and *u_m_*(*j*). The vectors distance $d_{i,j}^{m}=\max\left\{\left|{(u}_{m}\left(i+k\right){-\bar{u}}_{m}\left(i\right){)-(u}_{m}\left(j+k\right){-\bar{u}}_{m}\left(j\right))\right|,\;0\;\leq \;k\;\leq \;m-1\right\}$ gives the similarity degree between *u_m_*(*j*) to *u_m_*(*i*) that was added through the fuzzy function $D_{i,j}^{m}(n,r)=\mu \left(d_{i,j}^{m},,,n,,,r\right)=\exp(-{\left(d_{i,j}^{m}\right)}^{n}/r)$. Here *r* is the threshold parameter and *n* the exponent parameter.

Thus, the FE can be defined by Equation (3):

(3) $$FE(m,n,r,N)=\ln(\frac{1}{N-m}\overset{N-m}{\underset{i=1}{\sum }}(\frac{1}{N-m-1}\overset{N-m}{\underset{j=1,j\neq i}{\sum }}D_{i,j}^{m}(n,r)))-\ln(\frac{1}{N-m}\overset{N-m}{\underset{i=1}{\sum }}(\frac{1}{N-m-1}\overset{N-m}{\underset{j=1,j\neq i}{\sum }}D_{i,j}^{m+1}(n,r)))$$

FE introduces a sort of intermediate state proper of the fuzzy theory, assuming a “*membership degree*” for each point of the time series rather than a conventional two-state classifier as AE and SE [28]. Fuzzy Entropy and its multiscale version (MFE) found application in several studies concerning the HRV analysis of pathological subjects [17,45]. As in [29], we considered for all the scales an embedding dimension *m* = 2, *r* = 0.2 (of the time series standard deviation) and *n* = 2.

### 2.4. Permutation Entropy

From the time series {*X_i_*} = {*x*_1_…*x_N_*} of length *N*, consider (*N*−*m*−1) delay vectors *X_i_* = [*x_i_*…*x_i+_*_(*m*−1)_], where *m* is the embedding dimension. Each vector is rearranged in ascending order, thus for each delay vector there are *m!* possible reorder patterns *π_i_* (*i* = 1:*m!*), also called “motifs”. The relative frequency $p_{\pi_{i}}$ of the possible occurrences of the *i*-th motif *f*(*π_i_*) in the time series is given by:

(4) $$p_{\pi_{i}}=\frac{{f(\pi }_{i})}{N-m+1}$$

Thus, from [30], the Permutation Entropy (PE) can be expressed in terms of the Shannon Entropy, by:

(5) $$\;PE\left(m\right)=-\overset{m!}{\underset{\pi_{i}}{\sum }}p_{\pi_{i}}\log_{2}p_{\pi_{i}}$$

As for AE and SE, PE has several applications in HRV analysis and for seizure detection or characterization [17]. For all the PE scales, we considered the embedding dimensions *m* = 2 and *m* = 3 [30,46]. The multiscale version of PE (MPE) was already evaluated in previous ECG studies [46], showing its better capability to enhance differences between groups (pathological vs controls) than the single scale case.

### 2.5. Distribution Entropy

Distribution Entropy (DE) was proposed by Li et al. [31]. One of the main advantages of DE with respect to AE and SE, is its higher consistency in the complexity evaluation even for short-term RR signals. Similarly to SE, *m* is the embedding dimension and {*X_i_*} = {*x*_1_…*x_N_*} the original time series, where *N* is the number of samples. The distance $d_{i,j}^{m}=\max\left\{\left|u\left(i+k\right)-u\left(j+k\right)\right|,\;0\;\leq \;k\;\leq \;m-1\right\}$ among vectors *u_i_* and *u_j_* was computed. 

The $d_{i,j}^{m}$ are the entries of the distance matrix ${D=\{d}_{i,j}^{m}\}$. To calculate the distribution entropy, the empirical probability density function (ePDF) of ***D*** was estimated by the histogram approach, defining a priori the number of bins *B* and excluding the self-matches (*i.e., i = j*). Thus, the definition of Distribution Entropy using the formula of the Shannon Entropy [31] is shown in Equation (6):

(6) $$\mathrm{DistEn}\left(B\right)=-\overset{B}{\underset{t=1}{\sum }}p_{t}\log_{2}\left(p_{t}\right)\;$$

Therefore, the normalized Distribution Entropy [31] is defined in Equation (7):

(7) $$\mathrm{DE}\left(B\right)=-\frac{1}{\log_{2}\left(B\right)}\overset{B}{\underset{t=1}{\sum }}p_{t}\log_{2}\left(p_{t}\right)\;$$

where p*_t_*, *t =* 1…*B*, is the probability of each bin. Additionally, in this case, we considered an embedding dimension *m* = 2 and the number of bins *B* = 512 [31]. The multiscale version (MDE) of DE was evaluated in previous studies on ECG [47], showing less dependence on the time series length than MSE and MPE.

### 2.6. Statistical Analysis

This study aims at evaluating if HRV-entropy indexes allow discriminating between windows with seizure events and seizure-free ones. Specifically, it aims at discriminating between a newborn with seizures and a seizure-free one. We opted for two statistical analyses in order to evaluate general differences between the two populations.

The first analysis evaluated if a newborn with seizures might have different characteristics both for its ictal and interictal activities from a seizure-free patient. Then, we evaluated if some differences could be found between seizure windows and interictal ones, that might be helpful in the process of seizure detection [11].

The second analysis was performed to evaluate if a patient with seizures might have different characteristics than a seizure-free patient without distinguishing between seizure windows and interictal windows. In other words, we assessed whether the differences in HRV analysis are also found in the interictal activity and not only during seizures. This would support the hypothesis that neonatal seizures alter the cardio-regulatory system of the newborn not only during the ictal events, thus allowing the a priori discrimination between the two groups [11,14,16].

These two different statistical analyses have been computed for all the multiscale entropy indexes described above. In the following paragraphs, we describe the analyses, the tests used and the assumptions made for each of them.

The hypothesis of normality distribution has been checked through the Shapiro-Wilk test (SW, level of significance α = 0.05). As the normality hypothesis was not confirmed for some indexes (MPE, MDE) and some scales for the other indexes, we applied non-parametric tests. All tests have been performed between the 33 median observations of patients with seizure events (here we refer to class “1” for the windows with seizures and to class “int” for the interictal windows) and the 19 median observations of the seizure-free patients (here we refer to the seizure-free windows as class “0”).

The first analysis evaluated if differences among various combinations of the three classes (“0”—“1”—“int”) exist. To this aim, we defined a multiple comparison test (MCT), applying a Bonferroni multiple comparison post hoc correction. We used MCT information obtained by the Kruskal-Wallis test (significance level α = 0.05) between the median of the windows of all the patients, considering the test for the three classes “0”, “1” and “int” and related pairwise comparisons. We performed this test to evaluate if differences could be found simultaneously between windows with seizure events, seizure-free windows and interictal windows. In particular, the comparison between class “1” and class “0” evaluated the capability of entropy indexes to catch differences between a window with seizure events, i.e., a window from a pathological patient and a window of seizure-free patients. In Section 3 we reported the results of the pairwise comparisons referring to each test as the combination of class evaluated (i.e., “0 vs. 1” KW-Test, “int vs. 1” KW-Test, “0 vs. int” KW-Test).

For the second analysis, we performed a Mann-Whitney test (significance level α = 0.05) to evaluate if entropy indexes could discriminate between patients with seizure events and seizure-free ones. Here we considered the median of all windows (labelled as “1” and “int”) of the newborns with seizures against the median of windows of seizure-free newborns. The statistical results of all the entropy indexes considered for each scale (from 1 to 6) are shown in Section 3. We referred to this test as MW-Test.

## 3. Results

In this section, we reported all the statistical results obtained for each multiscale entropy index considered. For each index, a figure shows the multiscale entropy trends for the three classes considered, representing: the seizure-free trend (class “0”), the seizure trend (class “1”) and the interictal trend (class “int”). All tests were performed on 33 patients with seizures vs. 19 seizure-free patients. We did not find any statistically significant differences in the comparison “0 vs. int*”* with the Kruskal Wallis Test for all the entropy indexes; therefore, we did not report them in the related tables. Instead, we found differences for the remaining comparisons: “0 vs. 1” and “int vs. 1” for some multiscale entropy indexes, thus we reported them. We remark that all the KW-Test’s *p*-values were adjusted applying the Bonferroni correction.

**Figure 3:**
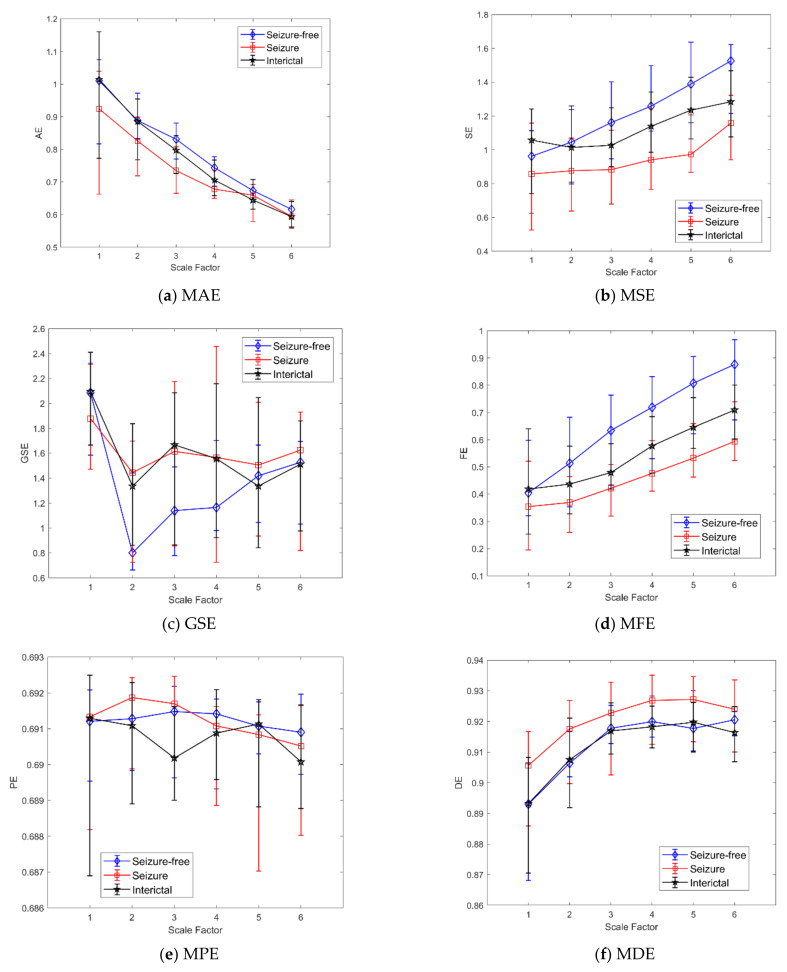
Comparison of the proposed approaches results for scales 1 to 6. (**a**) Approximate Multiscale Entropy. (**b**) Multiscale Sample Entropy. (**c**) Generalized Multiscale Sample Entropy. (**d**) Multiscale Fuzzy Entropy. (**e**) Multiscale Permutation Entropy. (**f**) Multiscale Distribution Entropy. The trends related to the seizure-free windows (◊ marker), seizure windows (□ marker) and interictal windows (☆ marker) are shown. The values at each scale represent the median and iqrs among patients.

Table 1 reports all the statistical analyses performed using MAE with embedding dimension *m* = 2 and a scale factor from 1 to 6 for multiscale analysis. Moreover, the statistics (median and IQR) for the three groups considered (0, 1, int) are included. In Figure 3a the MAE’s trends as a function of the scale factor are shown.

**Figure 4:**
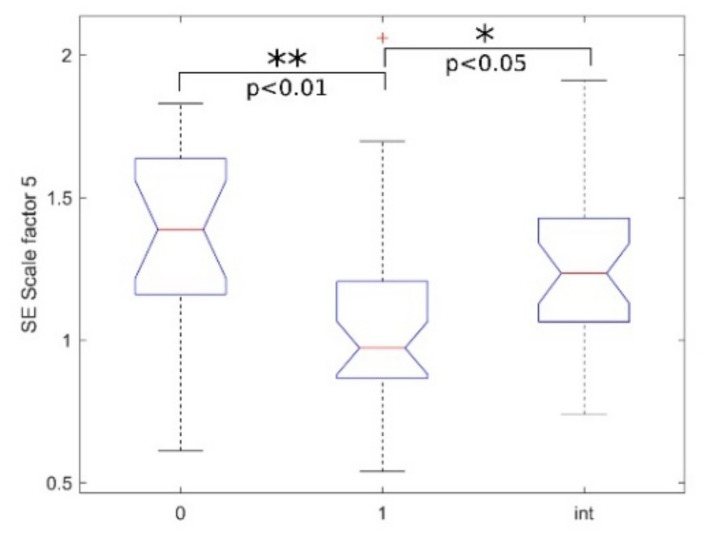
SE boxplots of the groups (0-1-int) with scale factor 5, where * and ** respectively denote statistically significant and highly statistically significant results obtained with the MCT test.

Table 2 reports the statistical analysis performed using MSE with embedding dimension *m* = 2 and a scale factor from 1 to 6 for multiscale analysis. The statistics (median and IQR) for the three classes considered are shown. In Figure 3b, the MSE trends as a function of the scale factor are shown. As we obtained a significant *p*-value in the “int vs. 1” KW-Test, Figure 4 shows the boxplot of the three classes at scale 5.

Table 3 reports the statistical analysis performed using GSE with embedding dimension *m* = 2 and a scale factor from 1 to 6 for multiscale analysis. At scale 1, the values are different from the MSE ones because the threshold factor *r* was set equal to 0.05 [28]. The statistics (median and IQR) for the three classes considered are included. In Figure 3c, the GSE trends as a function of the scale factor are shown.

**Figure 5:**
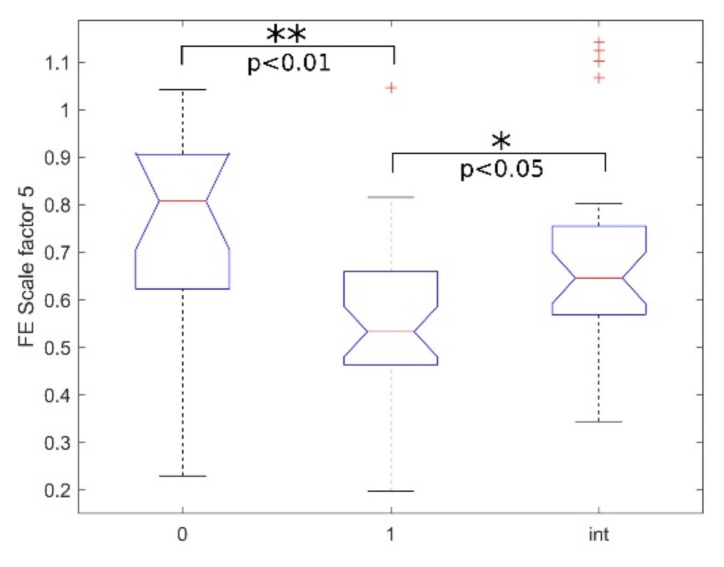
FE boxplot of the groups (0-1-int) with scale factor 5, where * and ** respectively denote statistically significant and highly statistically significant results obtained with the MCT test.

In Table 4 the statistical analysis performed using FE is reported, with embedding dimension *m* = 2 and a scale factor from 1 to 6 for multiscale analysis. As for MSE, the statistics (median and IQR) for the three classes considered are reported. In Figure 3d, the MFE trends as a function of the scale factor are shown. As we obtained a significant *p*-value in the “int vs. 1” KW-Test, Figure 5 shows the boxplot of the three classes at scale 5 and the corresponding multi-comparison analysis.

Table 5 reports the statistical analysis performed using PE with embedding dimension *m* = 2 and a scale factor from 1 to 6 for multiscale analysis. We also tested the embedding dimension *m* = 3. As the results were similar and therefore unnecessarily increasing the complexity of the model, they are not reported. Moreover, the statistics (median and IQR) for the three classes considered are reported. In Figure 3e, the MPE trends as a function of the scale factor are shown.

Table 6 reports the statistical analysis performed using DE with embedding dimension *m* = 2, number of bins 512 and a scale factor from 1 to 6 for multiscale analysis. As for MPE, the statistics (median and IQR) for the three classes considered are reported. In Figure 3f, the MDE trends as a function of the scale factor are shown.

In Table 7 we show the descriptive statistics (median and IQR) concerning the complexity index (CI) and the relative slope [48] of all the multiscale entropy indexes and the classes considered.

## 4. Discussion

This work applies multiscale entropy analysis to HRV dynamics with the aim of providing useful information about possible autonomic nervous system dysregulation occurring during seizures in newborns. 

Table 1 gives some significant differences between groups with MAE. However, the multiscale analysis shows the well-known limits of this metric in the analysis of short time series [17]: at higher scales, it was no longer possible to resolve differences (from scale 4 in Table 1, “0 vs. 1” KW-Test). Moreover, the MW-Test for all the scales considered shows that MAE might be not suitable to discriminate between a patient with seizures and a seizure-free one. The results reported in Table 2 and Table 4 show that SE and FE are capable of discriminating between a patient with seizure events from a seizure-free one (“0 vs. 1” KW-Test and MW-Test). The multiscale analysis proved to be crucial to detect these differences. In fact, without any scale factor (i.e., *s* = 1, Equation (1)), no differences were found among subjects. Instead, Table 2 and Table 4 show that windows with seizure events have lower values of SE and FE than those obtained for the interictal and seizure-free windows. This is partially in line with what was already found for neonatal seizures using entropy indexes in EEG analysis [49]. Furthermore, the results shown in Table 7 summarize what was found with the statistical analysis. As an example, the CI for MSE and MFE on windows with seizure events are on average lower than those in seizure-free windows (Mann-Whitney test, *p*-values < 0.05 with Bonferroni correction). Instead, for MPE or MAE the CI values are very similar among the classes (Mann-Whitney test, *p*-values > 0.05 with Bonferroni correction).

Thus, considering the results obtained in the “0 vs. 1” KW-Test and MW-Test for MSE and MFE (Table 2 and Table 4), it seems that differences between a newborn with seizures and a seizure-free one do not exist only during the ictal events but also during the whole interictal activity. These results seem to confirm that neonatal seizure events may produce a direct or indirect continuous alteration at the level of the cardio-regulatory system. Multiscale entropy indexes may detect these abnormal heart rate dynamics, probably connected to a reduced variability or transient decelerations in heart rate dynamics [50].

Moreover, a slight difference between the results obtained with FE and SE could indicate a higher accuracy for FE. Results showed in Table 2 and Table 4 highlighted that the MFE index was able to catch the differences on more scales than MSE, showing more consistency and intrinsic robustness against noise in the time series [51]. The MSE and MFE entropies showed promising results, thus suggesting that higher scale levels should be exploited using variants of MSE, such as Modified multiscale entropy or others [35,36].

GSE (Table 3) deserves a different discussion from MAE. The significant differences in volatility found by the MW-Test at scale factor 3 suggest a different behaviour between groups about instantaneous variability of HRV properties. As suggested by Costa et al. [28], this behavior might be related to possible abnormal heart dynamics during the cardiac cycle of activation and recovery (e.g., during depolarization and repolarization). However, the relationship between these findings and physiological dynamics is still an open issue, and further analysis is required. The obtained results suggest that a coarse-grained procedure based on the mean value should be preferred over variance-based methods. However, as stated by Costa et al. [28], further analysis about GSE should be made by increasing the window size or using different entropy variants [35]. 

**Figure 6:**
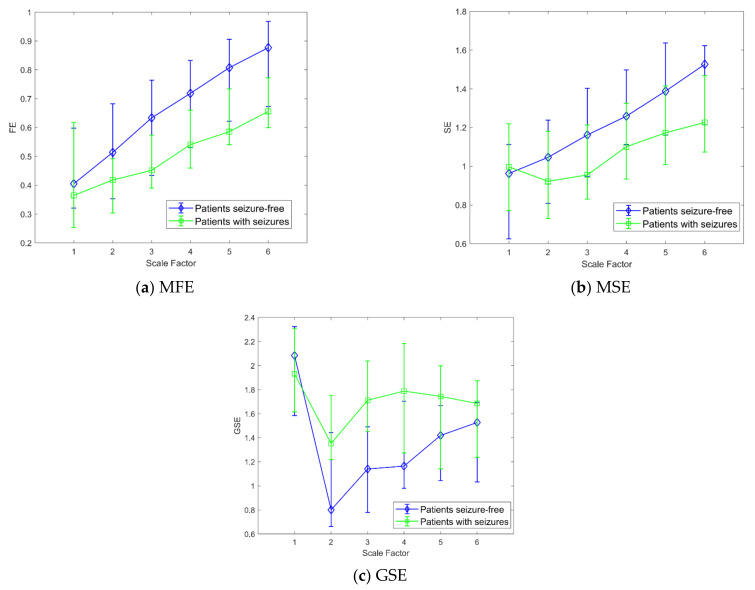
MFE (**a**), MSE (**b**), and GSE (**c**) trends considering all the windows for the patients with seizures (□ markers) compared to the seizure-free patients (◊ markers). The values at each scale represent the median and IQR among patients.

Concerning these findings, in Figure 6 the multiscale trends obtained for MW-Test (from Table 2, Table 3 and Table 4) are reported. The Figure shows that MFE, MSE and GSE were able to catch differences between seizures and seizure-free patients. Specifically, Figure 6 shows the cumulative trends related to patients with seizures for all the windows extracted, both those with seizure events (class “1”) and the interictal ones (class “int”), showing that differences still exist between the two groups. All the trends, starting from scale 2 for MFE and scale 3 for MSE, show that differences between a newborn with seizure and a seizure-free one can be found during or close to seizure events and during the interictal periods.

**Figure 7:**
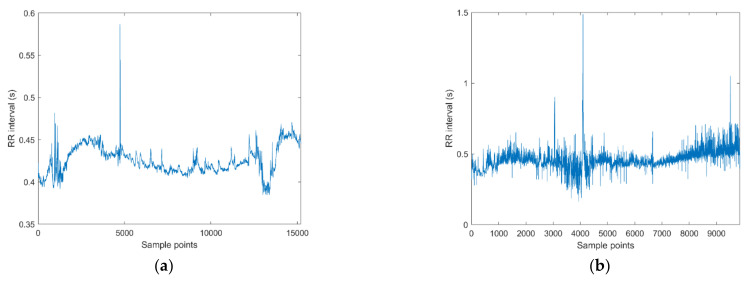
Examples of extracted inter-beat (RR) time series by Kubios: (**a**) newborn with seizure events; (**b**): seizure-free patient.

Figure 7 shows an example of two RR intervals trends: on the left, a patient with seizures, on the right a seizure-free patient. From a qualitative point of view, it can be noticed that the trend of the patient with seizures looks more regular than that of the seizure-free one. This is consistent with the results reported e.g., in Table 2 or Table 3 and Figure 6 (MSE, MFE), where the entropy values for a patient with seizures are on average lower than those of a seizure-free patient.

**Figure 8:**
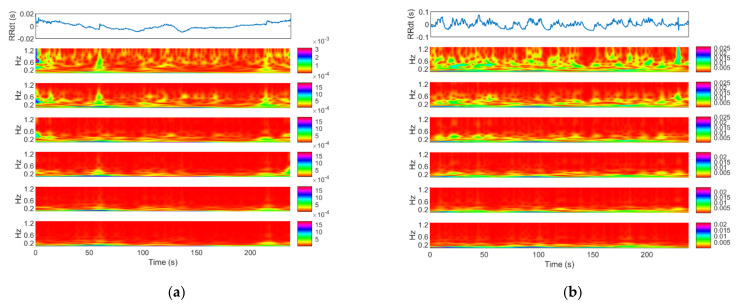
The multiscale wavelet power spectra of two 4-min length windows at different scales obtained by the coarse-grained scale (mean operator). (**a**): with seizure; (**b**): seizure-free. The first plot on the top, both for (**a**,**b**), is the original time series to which the coarse-grained scale was applied. Below, the wavelet power spectra from scale one to six are shown. The time-frequency representations were obtained by continuous wavelet transform. Frequency range: 0.04—1.3Hz.

To better explain how a multiscale analysis allows enhancing differences between time series, in Figure 8 six time-frequency representations by continuous wavelet transform (one for each scale) of two 4-min sequences are shown, one with seizure event (left picture) and one seizure-free (right picture): the higher the scale, the lower the magnitude of higher frequencies. Apart from the difference in magnitude, the contribution of low frequencies is preserved as expected. In this example, the difference between the two cases is marginal at scale 1 while they differ at higher scales. This is confirmed by statistical analysis. This picture qualitatively shows the usefulness of multiscale analysis instead of a single scale approach for the characterization of neonatal seizures.

Instead, as shown in Table 1, Table 5 and Table 6 for MAE, MPE, and MDE, we did not find any significant differences among groups. This suggests that, according to the used embedding dimension and threshold parameter, these indexes might not be helpful for HRV multiscale entropy analysis in newborns with seizures. As shown in the Results Section, differences between the methods considered here exist. Although an exhaustive answer about the possible reasons for all the entropies is challenging, it could be argued that the difference lies in the basic properties and limits of each method previously described. Limits of MAE and MSE have been discussed in [17,35]. Moreover, limits of MPE were found when applied to short time series, or with a low signal-to-noise ratio [52]. The present work focuses on a limited set of parameters and indexes and cannot be generalized, anyway it shows that the choice of the proper entropy index is crucial and requires a deeper analysis for a consistent and robust investigation.

Finally, although “0 vs. 1” KW-Test and MW-Test confirmed the differences between patients with seizures and seizure-free patients, we found that only MSE and MFE at scale factor 5 could discriminate between interictal windows and windows with seizure events (Table 2 and Table 4, “int vs. 1” KW-Test: *p*-value < 0.05). The result suggests that multiscale entropy indexes might not be suitable for an intra-patient analysis, an essential prerequisite for their implementation in a patient-independent ECG-based NSD [8,11]. However, this goal was out of the aims of this work and will be the subject of future studies.

In summary, our results confirm that HRV multiscale entropy analysis may provide helpful information for the characterization of neonatal seizures. As shown in [14], HRV analysis can provide a reliable marker for HIE, one of the most common neonatal seizures etiology.

Nevertheless, our analysis has some limitations: in general, the Helsinki Dataset includes a large number of newborns with episodes of asphyxia/HIE, that may have partly facilitated the implemented methods in differentiating between the groups. Moreover, our analysis did not cover extreme events such as sudden infant death syndrome (SIDS) that could be associated with an abnormal cardiac activity [15,53]. Thus, further studies are required to extend our findings to all the possible etiologies behind neonatal seizures. To the best of our knowledge, the Helsinki Dataset is the most extensive public dataset for neonatal seizures in terms of the number of patients [23], but we tested our methods on this dataset only. Thus, our findings may be considered preliminary and they need to be confirmed after their application to other datasets. This work does not consider all the entropy metrics proposed in the literature, thus the optimal entropy measures for this task will be the subject of further research. About MSE analysis, several variants were recently proposed [35] and will be considered as future developments of the methods, especially for the analysis of short time series. Promising methods, even for short time series, such as AvgApEn, AvgSampEn proposed by C. Karmakar et al. [54], ipApEn and ipSampEn introduced by G. Valenza et al. [55] may be valid alternatives to be evaluated.

Another issue concerns the choice of the most suitable window length. In this work, we used windows of 4 min of duration to detect autonomic variations in newborns [15]. This choice allowed for a consistent multiscale analysis and the discrimination between windows with seizure events and interictal windows. It is worthwhile noting that the choice of 4-min windows may not be the best and it might depend on the specific dataset. Thus, further studies would be focused on different datasets to confirm this finding. However, to detect the onset and the offset of an ictal event by HRV analysis, e.g., for a real-time evaluation, windows with shorter duration should be considered in future studies. 

Our results concern a single embedding dimension and a single threshold parameter. Although our settings are confirmed in the literature [17], an exhaustive research involving all the possible combinations of parameters, indexes and scales could be exploited. Moreover, a deeper analysis about parameters for multiscale entropy indexes could be taken into account in the future, in particular for indexes like PE and DE that were not able to detect the differences between newborns.

HRV entropy indexes seem to be appropriate to describe neonatal seizures with a specific etiology such as the hypoxic-ischemic encephalopathies or, in general, with asphyxia episodes [56,57]. Moreover, cardio-regulatory system differences from a newborn with seizures from a seizure-free one might also be present during interictal activity rather than only during or close to an ictal event.

## 5. Conclusions

This study concerned the evaluation of multiscale HRV entropy indexes for the characterization of neonatal seizures. Specifically, the capability of entropy measures to detect abnormal heart rate dynamics during or close to ictal events was exploited. Entropy measures were analyzed to discriminate between newborns with seizures and seizure-free ones. We found that Multiscale Sample and Fuzzy Entropy, from the scale factor 3 and 2 respectively, show significant differences between the two groups. Thus, the multiscale approach allows characterizing the ictal events that could not be detected with a single scale approach. Moreover, interictal activity showed significant differences between patients with seizure and seizure-free ones.

Though our results are promising, we are aware that HRV analysis may not be specific enough for neonatal seizure detection. For example, motor activity during seizures could lead to changes in heart rate and its variability, although neonatal seizures are often without a clear motor activity and can only be recognized from their electroclinical characteristics [1]. Thus, more studies are required to better clarify the neuro-vascular mechanisms occurring in a newborn with seizures and their relationships with multiscale entropy indexes.

In conclusion, the use of ECG and HRV multiscale entropy measures could be helpful as a pre-screening tool to quickly identify the newborns at high risk of seizures. Being easily implementable, minimally invasive, and cheap, this analysis could be successfully applied in clinical practice, mainly where EEG techniques are not available or not easily accessible.

## Figures and Tables

**Table 1:** Multiscale Approximate Entropy—Results of the test described in Section 2.6. The median and IQR values are reported for the three groups considered (0, 1, and int). Star (*) denotes statistically significant results.

MAE Scale Factor	0 vs. 1 KW-Test *p*-Value	int vs. 1 KW-Test *p*-Value	MW-Test *p*-Value	STATS 0 MEDIAN (IQR)	STATS 1 MEDIAN (IQR)	STATS INT MEDIAN (IQR)
1	0.4756	0.1252	0.8048	1.01 (0.82–1.07)	0.92 (0.66–1.04)	1.01 (0.77–1.16)
2	0.0809	0.2318	0.2312	0.88 (0.83–0.97)	0.83 (0.72–0.90)	0.89 (0.77–0.95)
3	0.0129 *	0.2160	0.0549	0.83 (0.77–0.88)	0.73 (0.67–0.81)	0.80 (0.73–0.84)
4	0.0937	0.7387	0.2872	0.74 (0.69–0.78)	0.68 (0.65–0.74)	0.71 (0.66–0.77)
5	0.7395	1	0.5686	0.67 (0.64–0.71)	0.66 (0.58–0.69)	0.64 (0.62–0.71)
6	1	1	0.6620	0.61 (0.56–0.64)	0.59 (0.56–0.65)	0.59 (0.56–0.64)

**Table 2:** Multiscale Sample Entropy—results of the test described in Section 2.6. Median and IQR values are reported for the three groups considered (0, 1, and int). Star (*) denotes statistically significant results.

MSE Scale Factor	0 vs. 1 KW-Test *p*-Value	int vs. 1 KW-Test *p*-Value	MW-Test *p*-Value	STATS 0 MEDIAN (IQR)	STATS 1 MEDIAN (IQR)	STATS INT MEDIAN (IQR)
1	1	0.1742	0.6213	0.96 (0.62–1.11)	0.85 (0.52–1.15)	1.05 (0.74–1.24)
2	0.1642	0.1733	0.3420	1.04 (0.80–1.23)	0.87 (0.63–1.06)	1.01 (0.79–1.25)
3	0.0054 *	0.0786	0.0503	1.16 (0.94–1.40)	0.88 (0.67–1.11)	1.02 (0.90–1.24)
4	0.0022 *	0.0990	0.0275 *	1.25 (1.11–1.49)	0.94 (0.76–1.24)	1.13 (0.98–1.34)
5	0.0022 *	0.0455 *	0.0318 *	1.38 (1.16–1.63)	0.97 (0.86–1.20)	1.23 (1.06–1.42)
6	0.0064 *	0.2387	0.0440 *	1.52 (1.21–1.62)	1.15 (0.94–1.32)	1.28 (1.07–1.46)

**Table 3:** Generalized Sample Entropy—statistical results of the test described in Section 2.6. The descriptive statistics (median and IQR) related to the three groups considered (0, 1, and int) are reported. Star (*) denotes statistically significant results.

GSE Scale Factor	0 vs. 1 KW-Test *p*-Value	int vs. 1 KW-Test *p*-Value	MW-Test *p*-Value	STATS 0 MEDIAN (IQR)	STATS 1 MEDIAN (IQR)	STATS INT MEDIAN (IQR)
1	1	0.4084	0.9092	2.08 (1.58–2.32)	1.87 (1.47–2.31)	2.09 (1.66–2.41)
2	0.5154	1.0000	0.0574	0.80 (0.66–1.44)	1.44 (0.72–1.69)	1.33 (0.86–1.83)
3	0.2617	1.0000	0.0166 *	1.14 (0.77–1.49)	1.61 (0.86–2.17)	1.66 (0.85–2.08)
4	0.9657	1.0000	0.0681	1.16 (0.97–1.70)	1.56 (0.72–2.45)	1.55 (0.92–2.15)
5	1	1.0000	0.0908	1.41 (1.04–1.66)	1.50 (0.93–2.00)	1.33 (0.84–2.04)
6	1	1.0000	0.1434	1.52 (1.03–1.69)	1.62 (0.82–1.93)	1.51 (0.97–1.85)

**Table 4:** Multiscale Fuzzy Entropy—Statistical results of the test described in Section 2.6. The descriptive statistics (median and IQR) related to the three groups considered (0, 1, and int) are reported. Star (*) denotes statistically significant results.

MFE Scale Factor	0 vs. 1 KW-Test *p*-Value	int vs. 1 KW-Test *p*-Value	MW-Test *p*-Value	STATS 0 MEDIAN (IQR)	STATS 1 MEDIAN (IQR)	STATS INT MEDIAN (IQR)
1	0.9564	0.2411	0.8344	0.40 (0.32–0.59)	0.35 (0.19–0.52)	0.41 (0.25–0.64)
2	0.0316 *	0.3318	0.0526	0.51 (0.35–0.68)	0.36 (0.25–0.46)	0.43 (0.32–0.57)
3	0.0052 *	0.2207	0.0150 *	0.63 (0.43–0.76)	0.42 (0.31–0.50)	0.47 (0.41–0.58)
4	0.0031 *	0.1444	0.0204 *	0.71 (0.53–0.83)	0.47 (0.41–0.59)	0.57 (0.47–0.68)
5	0.0013 *	0.0469 *	0.0194 *	0.80 (0.62–0.90)	0.53 (0.46–0.65)	0.64 (0.56–0.75)
6	0.0011 *	0.0864	0.0083*	0.87 (0.67–0.96)	0.59 (0.52–0.73)	0.70 (0.59–0.80)

**Table 5:** Multiscale Permutation Entropy—statistical results of the test described in Section 2.6. All tests were performed on 33 patients with seizures vs. 19 seizure-free patients. The descriptive statistics (median and IQR) related to the three groups considered (0, 1, and int) are reported.

MPE Scale Factor	0 vs. 1 KW-Test *p*-Value	int vs. 1 KW-Test *p*-Value	MW-Test *p*-Value	STATS 0 MEDIAN (IQR)	STATS 1 MEDIAN (IQR)	STATS INT MEDIAN (IQR)
1	1	1	0.7323	0.6912 (0.6895–0.6921)	0.6913 (0.6882–0.6925)	0.6913 (0.6869–0.6925)
2	0.5050	0.9519	0.3769	0.6913 (0.6898–0.6918)	0.6919 (0.6899–0.6924)	0.6911 (0.6889–0.6923)
3	1	0.3827	0.7467	0.6915 (0.6896–0.6922)	0.6917 (0.6890–0.6925)	0.6902 (0.6890–0.6916)
4	1	1	0.8792	0.6914 (0.6893–0.6918)	0.6911 (0.6889–0.6916)	0.6909 (0.6896–0.6921)
5	1	1	0.4529	0.6911 (0.6903–0.6918)	0.6908 (0.6870–0.6914)	0.6911 (0.6888–0.6918)
6	1	1	0.4359	0.6909 (0.6897–0.6920)	0.6905 (0.6880–0.6917)	0.6901 (0.6888–0.6917)

**Table 6:** Multiscale Distribution Entropy—statistical results of the test described in Section 2.6. The descriptive statistics (median and IQR) related to the three groups considered (0, 1, and int) are reported.

MDE Scale Factor	0 vs. 1 KW-Test *p*-Value	int vs. 1 KW-Test *p*-Value	MW-Test *p*-Value	STATS 0 MEDIAN (IQR)	STATS 1 MEDIAN (IQR)	STATS INT MEDIAN (IQR)
1	0.3664	0.2952	0.2312	0.8931 (0.8681–0.9062)	0.9057 (0.8859–0.9167)	0.8932 (0.8706–0.9083)
2	0.8091	0.5365	0.7323	0.9064 (0.9019–0.9168)	0.9176 (0.8998–0.9269)	0.9075 (0.8919–0.9211)
3	1	0.6727	1.0000	0.9178 (0.9128–0.9253)	0.9228 (0.9026–0.9328)	0.9169 (0.9094–0.9261)
4	1	0.2114	0.7756	0.9200 (0.9149–0.9283)	0.9269 (0.9125–0.9351)	0.9183 (0.9114–0.9250)
5	1	0.7985	0.8942	0.9177 (0.9104–0.9300)	0.9272 (0.9134–0.9347)	0.9198 (0.9100–0.9263)
6	1	0.3935	0.9697	0.9205 (0.9154–0.9233)	0.9240 (0.9100–0.9336)	0.9164 (0.9069–0.9249)

**Table 7:** Descriptive statistics (median and IQR) for all the entropy indexes and the classes considered (“0-1-int”) concerning the complexity index (CI) with the corresponding slope (+1 if positive −1 if negative). Stars (*) denote significant differences between class “0” and “1” (Mann-Whitney Test *p*-value < 0.05, after Bonferroni correction).

Multiscale Entropy Index	CI—Median (iqr) Class “0”	CI—Median (iqr) Class “1”	CI—Median (iqr) Class “INT”
MAE	−3.9 (−4.2: −3.7)	−3.6 (−3.9: −3.1)	−3.8 (−4.0: −3.4)
MSE *	6.3 (5.1: 7.2)	4.2 (2.6: 5.6)	5.0 (3.3: 6.2)
MFE *	3.1 (2.3: 3.7)	2.3 (1.7: 2.7)	2.6 (1.6: 3.1)
GSE	−5.9 (−8.1: −3.1)	−2.1 (−7.0: 9.4)	−5.7 (−8.2: −1.3)
MPE	−3.4 (−3.5: 3.5)	−3.4 (−3.5: 3.4)	−3.4 (−3.5: 3.4)
MDE	4.6 (4.5: 4.6)	4.6 (4.0: 4.6)	4.6 (4.5: 4.6)

## Data Availability

Data available on request, please contact the corresponding author at lorenzo.frassineti@student.unisi.it.
